# Does family-centred neonatal discharge planning reduce healthcare usage? A before and after study in South West England

**DOI:** 10.1136/bmjopen-2015-010752

**Published:** 2016-03-10

**Authors:** Jenny C Ingram, Jane E Powell, Peter S Blair, David Pontin, Maggie Redshaw, Sarah Manns, Lucy Beasant, Heather Burden, Debbie Johnson, Claire Rose, Peter J Fleming

**Affiliations:** 1 School of Social and Community Medicine, University of Bristol, Bristol, UK; 2 Faculty of Health and Applied Sciences, University of the West of England, Bristol, UK; 3 Faculty of Life Science and Education, University of South Wales, Pontypridd, UK; 4 Department of Population Health, NPEU, University of Oxford, Oxford, UK; 5 South West Neonatal Network, Bristol, UK; 6 Southmead Hospital, North Bristol NHS Trust, Bristol, UK

**Keywords:** Neonatal care, family-centred, self-efficacy, discharge planning

## Abstract

**Objective:**

To implement parent-oriented discharge planning (Train-to-Home) for preterm infants in neonatal care.

**Design:**

Before and after study, investigating the effects of the intervention during two 11-month periods before and after implementation.

**Setting:**

Four local neonatal units (LNUs) in South West England.

**Participants:**

Infants without major anomalies born at 27–33 weeks’ gestation admitted to participating units, and their parents.

**Train-to-Home intervention:**

A family-centred discharge package to increase parents’ involvement and understanding of their baby's needs, comprising a train graphic and supporting care pathways to facilitate parents’ understanding of their baby's progress and physiological maturation, combined with improved estimation of the likely discharge date.

**Main outcome measures:**

Perceived Maternal Parenting Self-Efficacy (PMP S-E) scores, infant length of stay (LOS) and healthcare utilisation for 8 weeks following discharge.

**Results:**

Parents reported that the Train-to-Home improved understanding of their baby's progress and their preparedness for discharge. Despite a lack of change in PMP S-E scores with the intervention, the number of post-discharge visits to emergency departments (EDs) fell from 31 to 20 (p<0.05), with a significant reduction in associated healthcare costs (£3400 to £2200; p<0.05) after discharge. In both study phases, over 50% of infants went home more than 3 weeks before their estimated date of delivery (EDD), though no reduction in LOS occurred.

**Conclusions:**

Despite the lack of measurable effect on the parental self-efficacy scores, the reduction in ED attendances and associated costs supports the potential value of this approach.

**Strengths and limitations of this study**

- This is the first study to measure the impact of a neonatal family-centred care intervention on parental self-efficacy or use of emergency department (ED) post-discharge for moderately preterm infants.
- Health economic data collection was available for most families which facilitated a detailed analysis of the costs of healthcare usage following discharge.
- The lack of time for implementing the Train-to-Home intervention meant that some staff were not confident in using the family-centred approach to discharge planning.
- The quasi-experimental study design (before and after) was also a limitation in that the changes in outcome measures were not randomised between units, but there were no significant differences in the infant or maternal demographics between the two study periods.

## Introduction

The improvements in survival of preterm infants over the past 20 years mean that more than 90% of infants born at 27 weeks’ or more gestation will survive to go home.^1^ For most infants, a relatively short period in a neonatal intensive care unit (NICU) will be followed by a longer period in high dependency and then special care before discharge home.^2^

Parents of preterm infants need to learn how to care for them after discharge home, and to prepare themselves and their home environment. Evidence suggests that ex-preterm infants make a disproportionate demand on emergency and ‘out-of-hours’ health services.^3^ ^4^ Parents have particularly expressed concern and uncertainty about how best to respond to minor illness or changes in routine for their babies.^5^ ^6^

A structured approach to discharge planning using care pathways and predictable timings for discharge improves the quality of care before and after discharge and reduces the need for unexpected re-admission after discharge, as well as improving patient satisfaction.^7^ ^8^ Parent-focused or family-centred neonatal care involves providing accurate information, and individualised care, including parents in infant care, and promoting positive relationships with staff.^9^ ^10^

Many parents of preterm infants are routinely informed by neonatal staff that their baby will be discharged home at or around the time the baby was due to be born—that is, the estimated date of delivery (EDD). This continues despite increasing evidence that improvements in neonatal care over recent years have led to shorter stays in hospital and earlier discharge to home.^11^ Using EDD as the expected discharge date means that, in many neonatal units, the process of preparing parents to take their baby home is often left until shortly before the baby is to be discharged. Many parents feel unprepared as a result and lack confidence to care for their baby.^6^ ^12^ ^13^

In an audit of the length of stay (LOS) of preterm infants in local neonatal units (LNUs; as defined by the UK Department of Health)^2^ in the Southwest region from 2011 to 2013, we found that almost all infants born at 27–33 weeks’ gestation were discharged home well before their original EDD, with almost 50% being discharged home around 4 weeks before this date.^14^ Manktelow also showed that infant LOS varies between neonatal units, so using local data may be helpful.^11^

Building on work from McMaster Children's Hospital, Canada,^15^ and using an extensive Delphi process with neonatologists, neonatal nurses and parents, we developed a UK parent pack (Train-to-Home) aimed at supporting parents’ preparedness to take their baby home. Use of the Train-to-Home encourages parents to participate in their baby's care from an early stage, to develop a fuller understanding of their baby's needs and the physiological maturation needed before babies can be discharged. The pack is parent centred and provides a practical means of improving communication between staff and parents throughout the baby's hospital stay. By improving parents’ self-confidence to care for their baby at home, we anticipated facilitating earlier discharge and reducing emergency or out-of-hours service use after discharge.

Neonatal care is an expensive and limited health resource with prematurely born infants occupying the majority of neonatal hospital bed-days.^16^ The average LNU cost in the UK is over £13 000 for each very low birthweight baby (birth weight <1500 g, which is the mean birth weight at 30 weeks’ gestation). Any increase in parental confidence to care for their infant could reduce their LOS, and possibly reduce healthcare resource use after discharge, making potentially significant healthcare savings.^17^

## Aim

The specific aims of the study were to investigate whether introducing the parent-centred neonatal discharge package (Train-to-Home) increased parental confidence in caring for their infant (self-efficacy), reduced infants’ length of hospital stay and reduced healthcare resource use after discharge from hospital.

## Train-to-Home intervention

**Figure 1:**
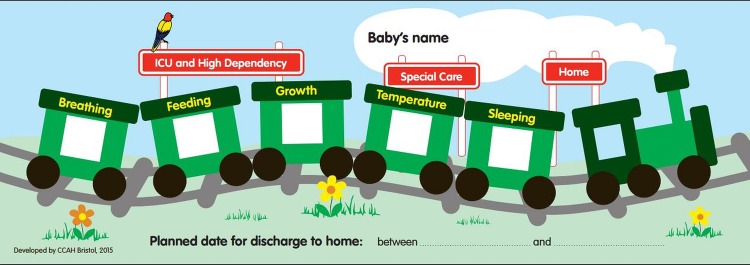
Train-to-Home.

**Figure 2:**
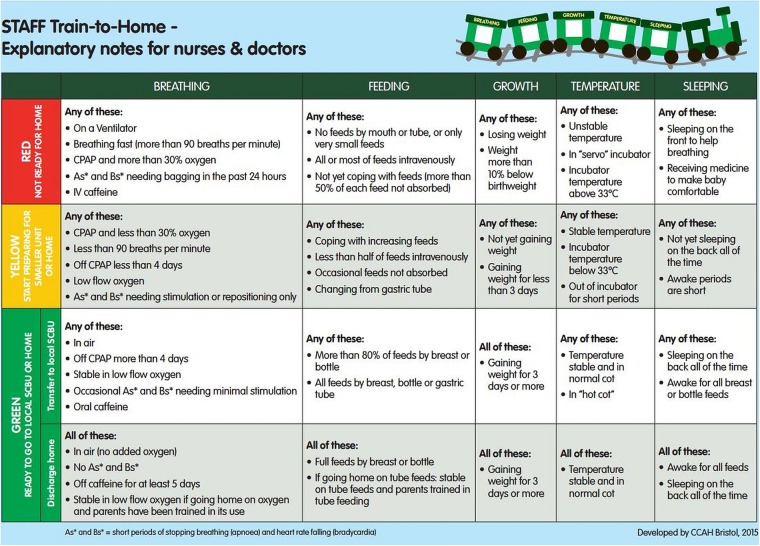
Explanation of the window colours on the Train-to-Home.

**Figure 3:**
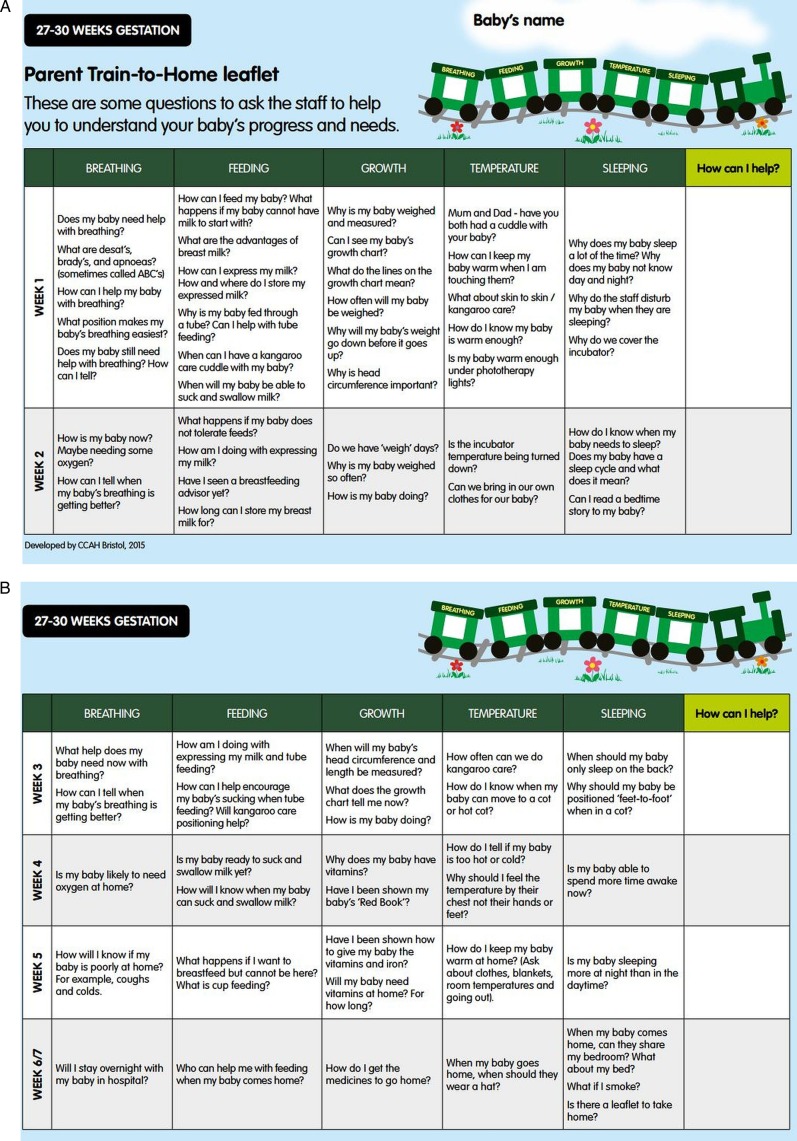
Parent Train-to-Home leaflet for 27–30 weeks’ gestation infants.

Soon after admission to the neonatal unit, an accurate estimate of when the baby is likely to be discharged from hospital is provided, based on the locally derived 50th–75th centiles for LOS for each gestation. The discharge date range is displayed on a laminated train which has five labelled carriages: breathing, feeding, growth, temperature and sleeping (figure 1). Using agreed criteria, parents change the carriage window sticker colour from red to yellow and then green to indicate the stage of preparedness for discharge home (figure 2). Parents are also given gestation-specific leaflets with questions linked to the five windows of the train to discuss with staff (figure 3) to help them understand their baby's progress and needs. Each week, the discharge date range narrows as the baby matures and a smaller range of dates is displayed as the baby approaches being ready for discharge. The Train-to-Home intervention was developed for use with all infants of gestational ages between 27 and 33 weeks in the target LNUs.

## Methods

### Study design and population

Parents of infants born between 27 weeks 0 days and 33 weeks 6 days were recruited in four large LNUs in South West England. Two of the units had associated level 1 units to which some infants were transferred before going home. Parents were recruited during two 11-month periods (phase 1: October 2012–August 2013 and phase 2: October 2013–August 2014) before and after the introduction in the LNUs of the Train-to-Home (with parent pathways). For the research study, infants with major congenital anomalies or with mothers under 16 years were not recruited. Parental assent was sought by the LNU nurses and consent gained by a study researcher.

### Outcome measures

Demographic and clinical information was collected by the researchers for all participating infants. Both parents were asked to complete a validated standardised measure, the Perceived Maternal Parenting Self-Efficacy (PMP S-E)^18^ tool, at three time points to measure perceived parental self-confidence when caring for their infant: soon after their baby's admission to the LNU, shortly before discharge home and 8 weeks after discharge.

A short healthcare resource use data collection tool was developed for parents to record all healthcare contacts for the baby, from which data were collected at telephone follow-up 4 and 8 weeks after discharge. Qualitative data were collected from parents 8 to 10 weeks following discharge by semi-structured telephone interviews exploring their experiences of the unit and perceptions of the intervention. All data were anonymised before analysis. Focus groups were also held to explore the views of nursing staff, and telephone interviews conducted with senior medical staff from all LNUs. Parent interviews and nursing staff focus groups were audio recorded, transcribed verbatim and analysed using thematic methods facilitated by the qualitative package NVivo.

Statistical analysis was performed using IBM SPSS Statistics V.21 and Stata V.13. For proportional data, χ^2^ tests were used to n−1 degrees of freedom. A test of normality on continuous data was conducted using the Shapiro-Wilk test and observing the Q–Q plots. The Mann-Whitney U test was used for non-parametric data, which were described using medians and IQRs. Resource use data in volume units were combined with price and unit cost information from published sources^19^ to estimate costs per item in £ sterling using 2014 prices. All cost variables were named and defined.

## Results

**Figure 4:**
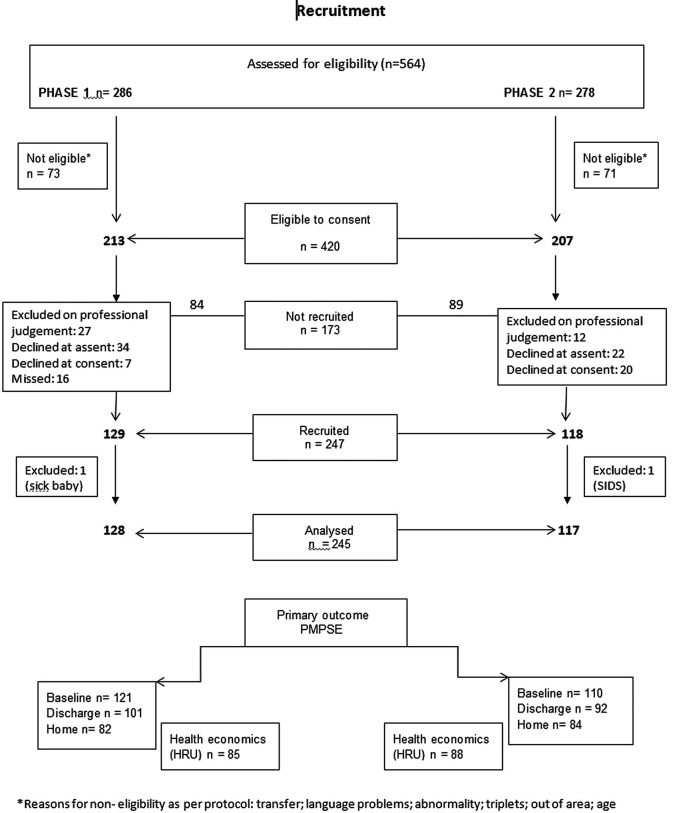
Train-to-Home study recruitment diagram.

A total of 245 families participated in the study, 128 families in phase 1 and 117 families in phase 2 as shown in figure 4. There were no significant demographic differences between infants and their families in the two phases as shown in tables 1 and 2. They were well matched for infant sex, gestation, birth weight, socioeconomic status, maternal conditions and breast versus bottle feeding. There was no difference in overall severity of illness or prevalence of cardiorespiratory or infective conditions between the groups, but metabolic, endocrine, gastroenterological and neurological problems were more common in infants in phase 1 as shown in table 3.

**Table 1:** Infant characteristics

Characteristic	Group of interest	Phase 1		Phase 2		p Value
		n/N	(%)	n/N	(%)	
Gender	Male	64/128	(50)	62/117	(54)	0.64
Twin	Yes	16/128	(13)	25/117	(21)	0.06
	**Units**	**Mean**	**(N, SD)**	**Mean**	**(N, SD)**	**p Value**
Birth weight	kg	1.70	(128, 0.50)	1.65	(114, 0.45)	0.44
Gestational age	Weeks/days	31 weeks 5 days	(128, 13 days)	31 weeks 4 days	(117, 12 days)	0.59

**Table 2:** Family demographics

Characteristic	Group of interest	Phase 1 n/N (%)	Phase 2 n/N (%)	p Value
Mother has partner	Yes	112/124 (90)	108/112 (97)	0.06
Maternal ethnicity	British	109/119 (92)	98/106 (93)	
	Other white*	2/119 (1.7)	5/106 (4.7)	0.18
	Other†	8/119 (6.7)	3/106 (2.8)	(2 df)
	**Units**	**Mean (N, SD)**	**Mean (N, SD)**	**p Value**
Maternal age	Years	30.7 (120, 5.7)	30.7 (111, 5.9)	0.98
Paternal age	Years	33.2 (77, 6.4)	32.5 (101, 6.8)	0.46
Deprivation score	IMD units	17.6 (126, 11.7)	16.2 (115, 12.4)	0.36

Maternal ethnicity: *British, Irish and any other white; †Indian, Pakistani, Caribbean, African, any other black and other.

**Table 3:** Infant medical conditions

	Phase 1		Phase 2		
Medical conditions	n/N	(%)	n/N	(%)	p Value
Cardiorespiratory conditions	46/128	(36)	39/117	(33)	0.67
Infections	26/128	(20)	25/117	(21)	0.84
Metabolic, endocrine, gastroenterological	28/128	(22)	12/117	(10)	0.01
Neurological	6/128	(4.7)	0/117	(0)	0.03*

*Using Fisher's exact test.

Table 4 shows the overall median maternal PMP S-E scores in phases 1 and 2 at baseline, discharge and 8 weeks after discharge. The scores were not significantly different between the phases. The median improvement in individual mothers’ scores between baseline and discharge home was slightly higher in phase 2 than in phase 1 (+14 vs+11), but this was not statistically significant (table 5). Similar findings were seen in the paternal PMP S-E scores. The improvements in maternal PMP S-E scores (from baseline to 8 weeks post discharge) were slightly, but not significantly, greater in LNUs in which staff fully engaged with the intervention as was apparent from their reported attitudes in the qualitative interviews.

**Table 4:** Median maternal PMP S-E scores at baseline, discharge and at home 8 weeks following discharge

When measured	Phase 1			Phase 2			p Value
	N	Median	IQR	N	Median	IQR	
Baseline	121	60.0	54.0–69.5	110	59.0	54.0–67.0	0.33
Discharge	101	70.0	61.5–76.5	92	69.0	64.0–74.8	0.77
At home	82	74.0	66.0–79.0	84	74.0	70.3–78.0	0.52

PMP S-E, Perceived Maternal Parenting Self-Efficacy.

**Table 5:** Median change in individual maternal self-efficacy scores from baseline to discharge and from baseline to 8 weeks after discharge home

Increase in maternal self-efficacy	Phase 1 (n=128)	Phase 2 (n=117)	p Value
Baseline to discharge	+7	+8	0.60
Baseline to at home	+11	+14	0.10

The length of hospital stay in phase 1 (median 28 days, IQR 19.5–43.5) was not significantly different (p=0.32) from phase 2 (median 32 days, IQR 20–46). Almost 75% of infants were discharged home on or before the initially estimated Train-to-Home discharge dates. More infants in phase 2 were discharged home being mixed fed with breast milk and formula and fewer were exclusively formula fed (table 6).

**Table 6:** Infant outcomes in phases 1 and 2: length of stay and type of feeding on discharge

	Phase 1 (n=128)	Phase 2 (n=117)	p Value
Length of stay (median)	28 days (IQR 19.5–43.5)	32 days (IQR 20–46)	0.32
Feeding on discharge	Breast feeding=44%	Breast feeding=44%	0.13 (2df)
	Bottle feeding=35%	Bottle feeding=26%	
	Mixed feeding=22%	Mixed feeding=31%	
Type of milk at discharge	Breast=57%	Breast=62%	0.65
	Formula=24%	Formula=19%	
	Both=20%	Both=20%	

Cost analysis of healthcare after discharge is based on 173 infants, 85/128 in phase 1 and 88/117 in phase 2, for whom sufficient information was provided (response rates of 66% in phase 1 and 75% in phase 2, respectively). There were significantly more attendances at emergency departments (EDs) by infants in phase 1 than in phase 2 (31 vs 20, respectively; p=0.03), with an associated significantly higher estimated cost in phase 1 than phase 2 (£3400 vs £2200, respectively; p=0.03). There was no difference between phases 1 and 2 in the number of hospital re-admissions, or hospital outpatient appointments attended by the infants after discharge, and no difference in primary care attendances (table 7).

**Table 7:** Health economic outcomes

	Phase 1(n=85)	Phase 2 (n=88)	p Value
Attendances at ED	31	20	0.03
Cost of ED attendances	£3400/patient	£2200/patient	0.03
Re-admission inpatient days	78 days	85 days	0.78
Outpatient appointments	115	117	0.34

ED, emergency department.

Results from the qualitative interviews with 37 parents and 24 staff are reported in detail in a separate paper (Ingram in preparation). Parents were overwhelmingly positive about the ‘Train-to-Home’ package and reported feeling better prepared for home in phase 2 than phase 1. Most found the Train-to-Home helpful in showing them visually that their baby was progressing and described feelings of being given hope and feeling in control. Mothers, fathers and siblings enjoyed using it. Medical and nursing staff generally agreed that the intervention materials were helpful in explaining a baby's physiological progress to parents, but some nursing staff had concerns that the estimated discharge dates were too optimistic. The materials were introduced over a 6-week period which was not long enough to embed the materials into each LNU, and without this period of normalisation, some staff were uncertain about using the package. However, staff in one unit were particularly positive about the Train-to-Home intervention and were keen for it to continue.

## Discussion

There was a small but significant reduction in out-of-hours ED visits and associated costs after the introduction of the Train-to-Home intervention. This was particularly notable in view of an increase in ED attendances nationally over this period, which coincided with the introduction of the ‘111 out-of-hours’ service, which encouraged more callers to attend ED. There was no significant difference in the changes in PMP S-E scores between the two phases of the study; however, the change was slightly greater and parents reported feeling more confident in phase 2. PMP S-E scores increased between admission and discharge of the babies in both study phases, indicating improved parental self-efficacy.

The predicted discharge dates helped parents to prepare for home. The ways that staff engaged with the materials when communicating with them helped them feel more confident, as well as having something visual to show their baby's progress and stage of physiological readiness. The questions in the leaflets encouraged parents to ask appropriate questions in a timely fashion to improve their knowledge and understanding. Monitoring compliance was difficult to measure but staff feedback and attitudes expressed in the interviews indicated that staff engagement was different between the units.

Others have shown that a risk factor for increased use of health services is the parents’ perception that their prematurely born infant is vulnerable.^6^ Parents’ concerns evolve as they move from the neonatal unit to home, and these may be addressed by providing timely discharge information, as was available through our parent pathway leaflets, and early anticipatory guidance to help build parental confidence as they move towards taking their baby home.^6^

There was no significant change in LOS in the LNU, although more than half the infants went home at least 3 weeks before the EDD in both phases. During phase 2, all the LNUs were working towards gaining full WHO/UNICEF Baby Friendly Initiative accreditation (http://www.unicef.org.uk/BabyFriendly) and were therefore encouraging and supporting mothers to go home breast feeding, as reflected by the increased proportion of infants receiving some breast milk at discharge in phase 2. Breastfeeding is more difficult for preterm babies and is often a reason for a longer stay while mothers and babies learn how to breastfeed.

Medical and nursing staff considered the Train-to-Home package fitted well with the NHS discharge planning initiative, but some nursing staff were reluctant to engage fully, expressing concern that the estimated discharge dates were too optimistic. These findings may reflect the limited time available within the study for effective implementation and cascade training of nursing staff. Discharge planning has been shown to work best when it is mutually shared by neonatal unit teams and families, so it is important to find ways of enhancing this process.^20^

The need for neonatal units to develop a more family-orientated approach to care has been highlighted in recent years. In a survey of neonatal family-centred policy and practice in the UK, Redshaw and Hamilton^10^ found considerable variation between neonatal units. They recommended the development of parent-friendly policies to provide a more positive neonatal experience for families. The Train-to-Home package gives parents clear information about their baby's physiological progress which helps them to understand their baby's needs and promotes positive relationships with staff as they discuss progress on a daily basis. These are the fundamental elements of ‘family-centred’ care. Recently, others have explored mothers’^12^ and nurses’^21^ perceptions of family-centred neonatal care. Finlayson *et al*^12^ found little to support family-centred care practice in NICUs and emphasised the importance of improving staff–mother interactions and facilitating mothers’ opportunities to be their baby's primary caregiver. Trajkovski *et al*^21^ identified that nurses need ongoing organisational support, guidance and education to assist them in delivering family-centred care effectively and the Train-to-Home pack appears to do this.

We are not aware of any other studies that have systematically attempted to assess the impact of a neonatal family-centred care intervention on parental self-efficacy or use of ED post-discharge for moderately preterm infants and suggest that the Train-to-Home can contribute to family-centred care, when staff engage with the approach.

Limitations of the study include the lack of time for implementing the Train-to-Home intervention which meant that some staff were not confident in using the family-centred approach to discharge planning. The quasi-experimental study design (before and after) was also a limitation but was felt to be the most appropriate design for implementing a complex intervention in neonatal care. The ‘before and after’ design meant that the intervention and any changes in outcome measures were not randomised between units, but we found no significant differences in the infant or maternal demographics between the two study periods. Our study was also limited to infants of 27–33 weeks’ gestation based in four LNUs. In the future, it would be important to implement the package on a network-wide basis to ensure equity so that infants transferred between units would all be using similar discharge planning packages. It also needs to include the wider range of gestational ages cared for in neonatal units so that staff can use it for all infants.

Although our initial primary outcome measure (PMP S-E score) did not show any significant differences between the groups, the improvement in preparedness for discharge home reported by the parents and the measured reduction in ED attendances with associated cost reduction suggest the intervention had significant benefits. This approach to educating and involving parents in the care and needs of preterm babies in hospital has potential value and warrants further study and more widespread adoption.
